# Supplementary material for: Modeling glioblastoma heterogeneity as a dynamic network of cell states
Source: Mol Syst Biol. 2021 Sep 16;17(9):e10105. doi: 10.15252/msb.202010105 (PMC8444284; doi:10.15252/msb.202010105)
Supplement: Supplementary file 6 — Source Data for Figure 5 [file MSB-17-e10105-s004.zip › Figure5A_sourcedata/GSEA_3017/nosigdb_stateE.GseaPreranked.1622795031093/GAROFANO_IAVARONE_GPM_SUBTYPE.html]

Details for gene set GAROFANO\_IAVARONE\_GPM\_SUBTYPE[GSEA]

|  || Dataset | state63017 |
| Phenotype | NoPhenotypeAvailable |
| Upregulated in class | na\_pos |
| GeneSet | GAROFANO\_IAVARONE\_GPM\_SUBTYPE |
| Enrichment Score (ES) | 0.68608075 |
| Normalized Enrichment Score (NES) | 5.3552628 |
| Nominal p-value | 0.0 |
| FDR q-value | 0.0 |
| FWER p-Value | 0.0 |
Table: GSEA Results Summary

  

Fig 1: Enrichment plot: GAROFANO\_IAVARONE\_GPM\_SUBTYPE      
 Profile of the Running ES Score & Positions of GeneSet Members on the Rank Ordered List

  

| PROBE | GENE SYMBOL | GENE\_TITLE | RANK IN GENE LIST | RANK METRIC SCORE | RUNNING ES | CORE ENRICHMENT || 1 | PMP22 |  |  | 0 | 1.393 | 0.0319 | Yes |
| 2 | CTGF |  |  | 2 | 1.276 | 0.0598 | Yes |
| 3 | CYR61 |  |  | 3 | 1.169 | 0.0866 | Yes |
| 4 | IGFBP5 |  |  | 4 | 1.015 | 0.1098 | Yes |
| 5 | CCL2 |  |  | 5 | 0.975 | 0.1320 | Yes |
| 6 | ANXA1 |  |  | 12 | 0.826 | 0.1438 | Yes |
| 7 | CD44 |  |  | 14 | 0.806 | 0.1611 | Yes |
| 8 | CEBPD |  |  | 15 | 0.791 | 0.1792 | Yes |
| 9 | CRYAB |  |  | 16 | 0.788 | 0.1972 | Yes |
| 10 | ANXA2 |  |  | 18 | 0.733 | 0.2128 | Yes |
| 11 | PRSS23 |  |  | 21 | 0.722 | 0.2269 | Yes |
| 12 | CNN3 |  |  | 24 | 0.702 | 0.2406 | Yes |
| 13 | LGALS3 |  |  | 25 | 0.697 | 0.2565 | Yes |
| 14 | IFITM3 |  |  | 26 | 0.682 | 0.2721 | Yes |
| 15 | LIMCH1 |  |  | 29 | 0.641 | 0.2844 | Yes |
| 16 | TAGLN2 |  |  | 32 | 0.621 | 0.2963 | Yes |
| 17 | BDNF |  |  | 34 | 0.610 | 0.3090 | Yes |
| 18 | NEDD9 |  |  | 38 | 0.590 | 0.3190 | Yes |
| 19 | EMP1 |  |  | 40 | 0.584 | 0.3311 | Yes |
| 20 | CALD1 |  |  | 41 | 0.580 | 0.3444 | Yes |
| 21 | PMEPA1 |  |  | 44 | 0.544 | 0.3545 | Yes |
| 22 | IFITM2 |  |  | 46 | 0.536 | 0.3655 | Yes |
| 23 | ARID5B |  |  | 47 | 0.533 | 0.3777 | Yes |
| 24 | FRMD4B |  |  | 48 | 0.532 | 0.3899 | Yes |
| 25 | S100A10 |  |  | 51 | 0.527 | 0.3996 | Yes |
| 26 | SERPINE1 |  |  | 52 | 0.526 | 0.4116 | Yes |
| 27 | MRC2 |  |  | 56 | 0.505 | 0.4196 | Yes |
| 28 | MMP2 |  |  | 57 | 0.503 | 0.4311 | Yes |
| 29 | PDP1 |  |  | 69 | 0.454 | 0.4285 | Yes |
| 30 | HSPA5 |  |  | 73 | 0.449 | 0.4352 | Yes |
| 31 | SERPINB6 |  |  | 77 | 0.440 | 0.4418 | Yes |
| 32 | UPP1 |  |  | 78 | 0.438 | 0.4518 | Yes |
| 33 | SYTL2 |  |  | 82 | 0.437 | 0.4582 | Yes |
| 34 | COL5A2 |  |  | 83 | 0.434 | 0.4681 | Yes |
| 35 | CD59 |  |  | 89 | 0.418 | 0.4718 | Yes |
| 36 | SHROOM3 |  |  | 95 | 0.410 | 0.4753 | Yes |
| 37 | CD99 |  |  | 107 | 0.389 | 0.4712 | Yes |
| 38 | TPM1 |  |  | 108 | 0.388 | 0.4801 | Yes |
| 39 | LGALS1 |  |  | 113 | 0.368 | 0.4838 | Yes |
| 40 | FLOT1 |  |  | 114 | 0.368 | 0.4922 | Yes |
| 41 | FNDC3B |  |  | 123 | 0.354 | 0.4908 | Yes |
| 42 | AHNAK |  |  | 125 | 0.351 | 0.4977 | Yes |
| 43 | SEMA3A |  |  | 128 | 0.349 | 0.5033 | Yes |
| 44 | DSTN |  |  | 129 | 0.348 | 0.5113 | Yes |
| 45 | ARL6IP5 |  |  | 134 | 0.345 | 0.5145 | Yes |
| 46 | IRS1 |  |  | 140 | 0.342 | 0.5164 | Yes |
| 47 | SOCS3 |  |  | 141 | 0.342 | 0.5242 | Yes |
| 48 | NRCAM |  |  | 144 | 0.339 | 0.5296 | Yes |
| 49 | CETN2 |  |  | 145 | 0.338 | 0.5373 | Yes |
| 50 | PDLIM2 |  |  | 155 | 0.329 | 0.5342 | Yes |
| 51 | SPATS2L |  |  | 163 | 0.323 | 0.5334 | Yes |
| 52 | FZD7 |  |  | 164 | 0.323 | 0.5408 | Yes |
| 53 | DCBLD2 |  |  | 165 | 0.322 | 0.5481 | Yes |
| 54 | TFPI2 |  |  | 166 | 0.322 | 0.5555 | Yes |
| 55 | MBNL2 |  |  | 173 | 0.316 | 0.5556 | Yes |
| 56 | ATXN1 |  |  | 174 | 0.315 | 0.5628 | Yes |
| 57 | ARHGAP29 |  |  | 175 | 0.314 | 0.5700 | Yes |
| 58 | S100A6 |  |  | 176 | 0.312 | 0.5771 | Yes |
| 59 | A2M |  |  | 184 | 0.309 | 0.5759 | Yes |
| 60 | NME3 |  |  | 185 | 0.308 | 0.5830 | Yes |
| 61 | MAP1LC3A |  |  | 186 | 0.308 | 0.5900 | Yes |
| 62 | TGFB2 |  |  | 189 | 0.303 | 0.5946 | Yes |
| 63 | FLNA |  |  | 197 | 0.298 | 0.5932 | Yes |
| 64 | RUNX2 |  |  | 199 | 0.296 | 0.5987 | Yes |
| 65 | CAST |  |  | 202 | 0.294 | 0.6031 | Yes |
| 66 | CSF1 |  |  | 203 | 0.293 | 0.6098 | Yes |
| 67 | DKK3 |  |  | 205 | 0.293 | 0.6153 | Yes |
| 68 | SAT1 |  |  | 206 | 0.291 | 0.6220 | Yes |
| 69 | GNG12 |  |  | 212 | 0.287 | 0.6227 | Yes |
| 70 | CHPT1 |  |  | 216 | 0.284 | 0.6256 | Yes |
| 71 | PLEKHG1 |  |  | 217 | 0.284 | 0.6321 | Yes |
| 72 | TBX2 |  |  | 221 | 0.282 | 0.6350 | Yes |
| 73 | SDC2 |  |  | 226 | 0.280 | 0.6367 | Yes |
| 74 | PRRX1 |  |  | 229 | 0.278 | 0.6407 | Yes |
| 75 | TMED9 |  |  | 234 | 0.272 | 0.6422 | Yes |
| 76 | GPC4 |  |  | 246 | 0.264 | 0.6353 | Yes |
| 77 | CSRP2 |  |  | 247 | 0.264 | 0.6413 | Yes |
| 78 | TRIM56 |  |  | 248 | 0.263 | 0.6473 | Yes |
| 79 | CKAP4 |  |  | 250 | 0.262 | 0.6521 | Yes |
| 80 | PGM2L1 |  |  | 255 | 0.260 | 0.6534 | Yes |
| 81 | TRAM2 |  |  | 256 | 0.259 | 0.6593 | Yes |
| 82 | LEPROT |  |  | 259 | 0.259 | 0.6629 | Yes |
| 83 | SPP1 |  |  | 261 | 0.257 | 0.6676 | Yes |
| 84 | TNFRSF12A |  |  | 262 | 0.257 | 0.6734 | Yes |
| 85 | WWTR1 |  |  | 263 | 0.256 | 0.6793 | Yes |
| 86 | MOXD1 |  |  | 268 | 0.252 | 0.6803 | Yes |
| 87 | CHPF |  |  | 269 | 0.251 | 0.6861 | Yes |
| 88 | TUBA1C |  |  | 282 | -0.253 | 0.6777 | No |
| 89 | NUP37 |  |  | 286 | -0.255 | 0.6800 | No |
| 90 | YME1L1 |  |  | 327 | -0.264 | 0.6389 | No |
| 91 | HIST1H2BC |  |  | 332 | -0.264 | 0.6402 | No |
| 92 | TRIOBP |  |  | 388 | -0.278 | 0.5817 | No |
| 93 | RECQL |  |  | 390 | -0.279 | 0.5869 | No |
| 94 | CHRAC1 |  |  | 435 | -0.294 | 0.5417 | No |
| 95 | MRPS6 |  |  | 641 | -0.382 | 0.3087 | No |
| 96 | TTF2 |  |  | 646 | -0.386 | 0.3128 | No |
| 97 | SLC20A1 |  |  | 717 | -0.444 | 0.2404 | No |
| 98 | GSTO1 |  |  | 731 | -0.459 | 0.2356 | No |
| 99 | ANLN |  |  | 860 | -0.733 | 0.1014 | No |
Table: GSEA details [plain text format]

  

Fig 2: GAROFANO\_IAVARONE\_GPM\_SUBTYPE: Random ES distribution      
 Gene set null distribution of ES for **GAROFANO\_IAVARONE\_GPM\_SUBTYPE**
